# Uptake and effect of universal test-and-treat on twelve months retention and initial virologic suppression in routine HIV program in Kenya

**DOI:** 10.1371/journal.pone.0277675

**Published:** 2022-11-22

**Authors:** Davies O. Kimanga, Violet A. Oramisi, Amin S. Hassan, Mary K. Mugambi, Frederick O. Miruka, Kennedy J. Muthoka, Jacob O. Odhiambo, Peter K. Yegon, Gonza O. Omoro, Catherine Mbaire, Kenneth M. Masamaro, Susan M. Njogo, Joseph L Barker, Catherine N. Ngugi

**Affiliations:** 1 Division for Global HIV & TB, US Centres for Disease Control and Prevention, Nairobi, Kenya; 2 National AIDS and STI Control Program, Ministry of Health, Nairobi, Kenya; 3 HIV and STI Department, KEMRI/Wellcome Trust Research Programme, Kilifi, Kenya; 4 Epidemiology and Data Use, Palladium Group, Nairobi, Kenya; 5 Health Population and Nutrition, United States Agency for International Development, Nairobi, Kenya; 6 Strategic Information, Military HIV Research Program/Walter Reed Army Institute of Research, Nairobi, Kenya; 7 PEPFAR Coordinating Office, US Embassy, Nairobi, Kenya; University of the Witwatersrand, SOUTH AFRICA

## Abstract

Early combination antiretroviral therapy (cART), as recommended in WHO’s universal test-and-treat (UTT) policy, is associated with improved linkage to care, retention, and virologic suppression in controlled studies. We aimed to describe UTT uptake and effect on twelve-month non-retention and initial virologic non-suppression (VnS) among HIV infected adults starting cART in routine HIV program in Kenya. Individual-level HIV service delivery data from 38 health facilities, each representing 38 of the 47 counties in Kenya were analysed. Adults (>15 years) initiating cART between the second-half of 2015 (2015HY2) and the first-half of 2018 (2018HY1) were followed up for twelve months. UTT was defined based on time from an HIV diagnosis to cART initiation and was categorized as same-day, 1–14 days, 15–90 days, and 91+ days. Non-retention was defined as individuals lost-to-follow-up or reported dead by the end of the follow up period. Initial VnS was defined based on the first available viral load test with >400 copies/ml. Hierarchical mixed-effects survival and generalised linear regression models were used to assess the effect of UTT on non-retention and VnS, respectively. Of 8592 individuals analysed, majority (n = 5864 [68.2%]) were female. Same-day HIV diagnosis and cART initiation increased from 15.3% (2015HY2) to 52.2% (2018HY1). The overall non-retention rate was 2.8 (95% CI: 2.6–2.9) per 100 person-months. When compared to individuals initiated cART 91+ days after a HIV diagnosis, those initiated cART on the same day of a HIV diagnosis had the highest rate of non-retention (same-day vs. 91+ days; aHR, 1.7 [95% CI: 1.5–2.0], p<0.001). Of those included in the analysis, 5986 (69.6%) had a first viral load test done at a median of 6.3 (IQR, 5.6–7.6) months after cART initiation. Of these, 835 (13.9%) had VnS. There was no association between UTT and VnS (same-day vs. 91+ days; aRR, 1.0 [95% CI: 0.9–1.2], p = 0.664). Our findings demonstrate substantial uptake of the UTT policy but poor twelve-month retention and lack of an association with initial VnS from routine HIV settings in Kenya. These findings warrant consideration for multi-pronged program interventions alongside UTT policy for maximum intended benefits in Kenya.

## Introduction

Benefits of early combination antiretroviral therapy (cART) as a public health intervention in the management of HIV infection are indisputable. Early cART has been associated with reduced HIV associated morbidity and mortality [1–4]. With good adherence, early cART has also been shown to attain rapid and sustained HIV plasma virologic suppression [1, 4–7] and consequently associated with reduced risk of onward HIV transmission at the individual-level, household-level and population-level [8–10]. Benefits conferred by early cART informed the development of the 2015 WHO guidelines recommending immediate initiation of cART to all HIV infected individuals regardless of their clinical, immunological or virological status, commonly referred to as the universal ‘test-and-treat’ (UTT) policy [11].

If well implemented, the UTT policy may play a pivotal role in attaining the UNAIDS 95-95-95 targets toward ending the epidemic by 2030 [12]. By mid-2019, an estimated 93% of low- and middle-income countries (LMIC) had adopted the UTT policy [13]. However, as countries scale-up the UTT policy, uncertainties around preparedness of newly HIV diagnosed individuals for immediate cART, adherence to early treatment, sustained virologic suppression and un-interrupted engagement to long-term care are emerging [14–19].

A pooled meta-analysis of four randomized controlled trials (RCTs) reported increased virologic suppression and improved 12-months retention amongst individuals on rapid cART initiation (defined as cART initiation within 14 days of HIV diagnosis) [16]. Similarly, a Cochrane review of seven RCTs carried out in LMIC reported better rapid cART uptake, improved retention and better virologic suppression at 12-months amongst participants on rapid cART initiation (defined as cART offered within seven days of a HIV diagnosis) compared to standard care. Notably, in all seven RCTs, the reviewers observed that ‘…rapid ART was offered as part of a package that included several co-interventions targeting individuals, health workers and health system processes that aimed to facilitate uptake and adherence to ART’ [20]. More recently, data from individuals followed up in two RCTs in South Africa found no evidence that same-day ART initiation led to greater attrition or lower rates of viral suppression one year after starting ART [21].

While data from controlled clinical trial settings report better early cART uptake, improved retention and higher virologic suppression amongst individuals started on immediate cART compared to delayed cART, evidence from observational studies are conflicting. A pooled meta-analysis of eleven observational cohorts reported a tendency towards increased risk of being lost to follow up (LTFU) and a decreased likelihood of virologic suppression amongst individuals on rapid or early cART [16]. Furthermore, historical experiences from the implementation of immediate cART in pregnant women under the option B+ program cautioned poor initial retention as a challenge [22, 23].

Importantly, there is a dearth of evidence to describe uptake and effect of early cART on retention and virologic suppression in routine programmatic settings from LMIC. Data from 32 rural and urban facilities from a district in Malawi reported 6% higher 12-month retention after the introduction of UTT compared to the pre-UTT period [24]. Conversely, data from a programmatic pilot of UTT from 32 local government areas of Nigeria report 34% lost to follow up at 12-months after UTT (defined as starting cART within two weeks of HIV diagnosis) and low documented viral load monitoring of 8%. Of those with viral load results, 78% had achieved virologic suppression (<400 copies/ml) at 12-months of follow up, thus concluding that the effectiveness of UTT in some settings may be far lower than the efficacy demonstrated in randomized controlled trials [25]. More recently, data from two primary health clinics in South Africa reported higher on-ART attrition after the introduction of same-day ART initiation policy, compared to the pre-UTT period [26].

Thus, while early cART has been shown to improve retention and virologic suppression in controlled clinical trials, evidence from observational studies are conflicting. Much less is known about the uptake of UTT and its effect on retention and virologic suppression in routine ‘real life’ program settings. We aimed to describe the uptake and effect of UTT on twelve-month retention and initial virologic suppression amongst HIV infected individuals starting cART in a routine HIV program setting in Kenya.

## Methods

### Study design

A longitudinal analysis of data archived at the national data warehouse (NDW) was done. In brief, the NDW is a repository of individual-level routine HIV program data hosted at the National AIDS and STI Control Program (NASCOP) site in Nairobi, Kenya. The NDW infrastructure consists of electronic records stemming from health facilities offering HIV care and treatment services in the country and documenting the same in electronic medical record (EMR) systems. Routinely collected service delivery data from EMR systems are periodically auto-extracted and uploaded onto the NDW. Data uploaded onto the NDW undergoes de-duplication, de-identification and generation of a patient key value identifier using a data warehouse application programming interphase (DWAPI). By the end of 2019, the NDW hosted individual level longitudinal data from ~1.7 million HIV-infected individuals ever enrolled into an EMR system from 1,209 facilities covering 44 of the 47 counties in Kenya.

In Kenya, the UTT policy was implemented from July 2016. The policy recommends initiation of cART for all HIV-infected individuals as soon as possible, but preferably within two weeks of an HIV diagnosis. For the purpose of this analysis, data from HIV infected adults (age, >15 years old) starting cART between July 2015 –June 2018 were considered eligible. Individuals starting cART during the year preceding UTT implementation were included in the analysis to serve as a reference population. All individuals were followed up for 12-months after cART initiation.

### Sampling strategy

While routine service delivery data holds tremendous potential towards assessing impact of programmatic interventions in real-life settings, an innate challenge includes incomplete and/or missing data. For this reason, a convenient sampling approach was used. In brief, of the 1209 facilities transmitting data to the NDW, we sampled one facility from each of the 47 counties in Kenya based on the following criteria: (i) facilities with >50 and <1000 individuals starting cART during the study period (to control for under- or over-representation of the study population at the county level), and (ii) facilities that uploaded EMR data to the NDW after June 2019 (to provide an equal opportunity for at least 12-months of follow up time), and (iii) facilities with the lowest proportion of individuals with missing HIV diagnosis date and viral load test (as informed by the analysis endpoints). Thirty-eight facilities, representing 38 counties, were eligible (S1 Table). All individuals in eligible facilities were included in the analysis. Of the nine counties that were not included in the analysis, some had facilities that had never uploaded data to the NDW (n = 3), had facilities that had their last EMR data upload to the NDW before June 2019 (n = 6).

### Definition of indicators

Uptake of UTT was defined as time from the date of an HIV positive diagnosis to the date of cART initiation, categorized as follows: same day, 1–14 days, 15–90 days and 91+ days. Non-retention was defined as individuals who were either reported dead or determined LTFU by the end of the twelve-months follow up period. LTFU was defined as individuals missing a clinic visit more than 3 months after their last clinic appointment date. Individuals who transferred care to other facilities were included in the analysis and follow-up time censored at the last clinic visit date before the transfer. Initial virologic non-suppression (VnS) was determined based on the first available viral load test done within 12-months of cART initiation and defined as individuals with HIV RNA of >400 copies/ml.

Other indicators included gender, age at cART initiation, initial ART regimen, baseline CD4 T-cell count and calendar half-year of cART initiation. Initial ART regimen was defined based on antiretroviral drugs prescribed at the date of cART initiation. Baseline CD4 T-cell count was determined based on documented results from tests done within 6 months prior to cART initiation. Calendar half-year (HY) of cART initiation was defined based on the date of cART initiation and was categorized as follows: Jul-Dec 2015 (second-half of 2015; 2015HY2), Jan-Jun 2016 (first-half of 2016: 2016HY1), Jul-Dec 2016 (second-half of 2016: 2016HY2), Jan-Jun 2017 (first-half of 2017: 2017HY1), Jul-Dec 2017 (second-half of 2017: 2017HY2) and Jan-Jun 2018 (first-half of 2018: 2018HY1).

### Data analysis

Descriptive statistics were used to describe the study population. Proportion (95% confidence intervals, CI) of UTT uptake, non-retention and VnS were presented. Because same-day HIV diagnosis and cART initiation was not a rare endpoint, univariable and multivariable generalized linear models (glm) were to identify factors associated with UTT uptake. Hierarchical mixed effects modeling was also applied to control for within-and-between health facility (and county) variations. Crude and adjusted risk ratios (RR), 95% CIs and p-values were reported.

Time-to-event analysis was applied to determine time from cART to non-retention over a 12-months follow up period and was presented using Kaplan Meier survival curves stratified by UTT uptake. Non-retention rates at 12-months were reported per 100 person-months observations (pmo). Univariable and multivariable hierarchical mixed effects survival models were applied to determine the independent effect of UTT on non-retention after controlling for within-and-between health facility (and county) variations. Crude and adjusted hazard ratios (HR), 95% CIs and p-values were reported.

The analysis was then restricted to individuals with initial viral load test results (based on the first viral load test done within 12 months of cART initiation). Proportions (95% CI) of individuals with initial VnS stratified by UTT uptake were presented. Similarly, and because VnS was not a rare endpoint, univariable and multivariable hierarchical mixed effects generalized linear models were applied to determine the independent effect of UTT on initial VnS after controlling for within-and-between facility (and county) variations. Crude and adjusted risk ratios (RR), 95% CIs and p-values were reported.

### Ethical consideration

This retrospective analysis was considered part of a National HIV program evaluation exercise using routinely collected service delivery data archived at the NDW repository. As such, it was not possible to obtain informed consent from individuals. Rather, ethics approval to use data from the repository and to waive the need for informed consenting was obtained from the Africa Medical Research Foundation (AMREF) Ethics Review Committee, Kenya (AMREF-ESRC P716/2019). The analysis was also reviewed in accordance with the U.S. Centers for Disease Control and Prevention (CDC) human research protection procedures and was determined to be non-research. The investigators did not interact with human subjects or have access to identifiable data or specimens for research purposes. All the data shared for analysis were fully anonymized.

## Results

### Characteristics of study participants

**Fig 1:**
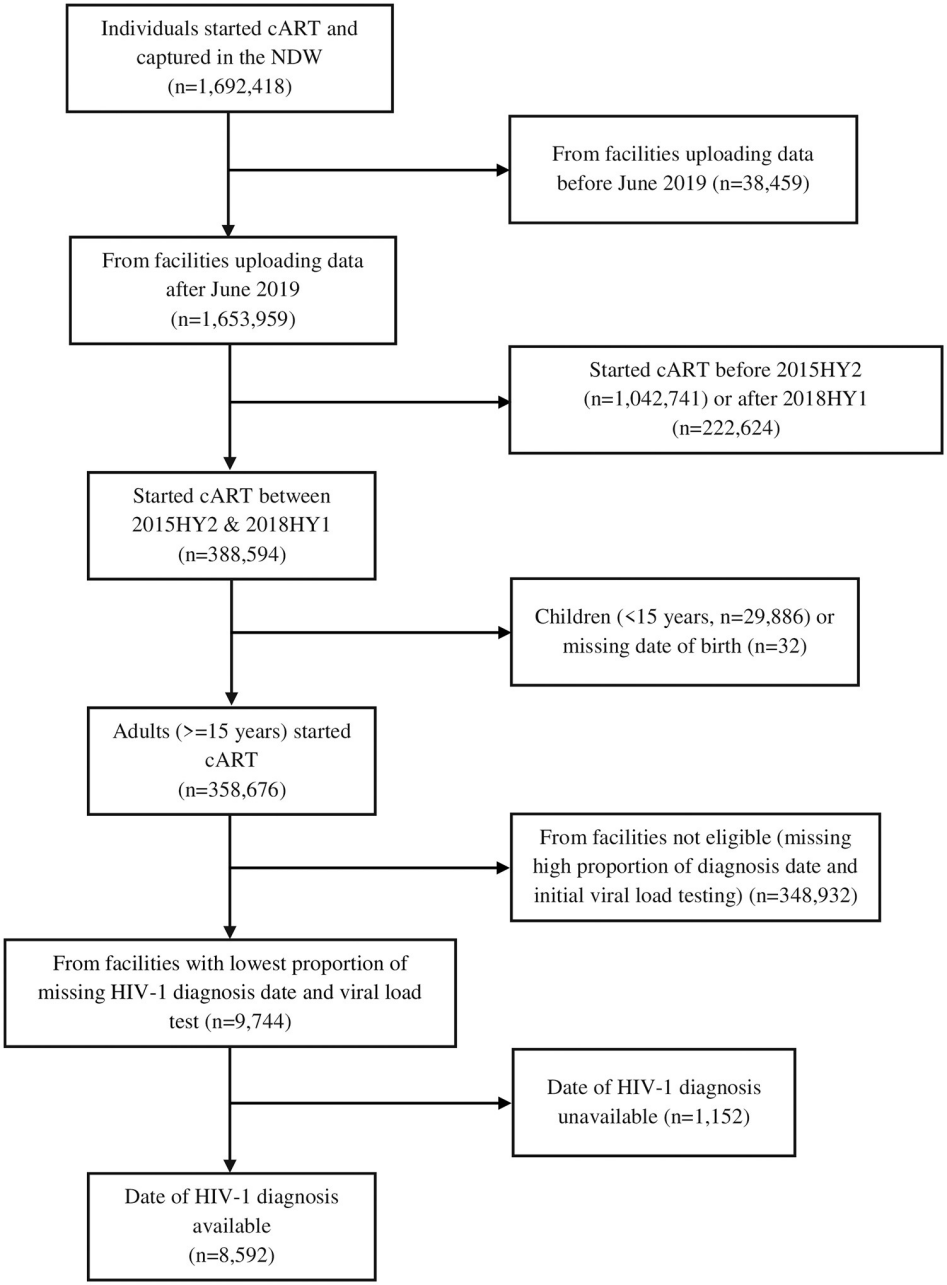
Flow chart showing distribution of HIV infected individuals captured in the national data warehouse (NDW) and selection of eligible individuals carried forward to the analysis.

Of the 1,692,418 individuals ever initiated on cART in Kenya and captured in the NDW, about 358,676 (here-with referred as the general population) were adults started on treatment between 2015HY2 and 2018HY1 and 9744 were sampled from facilities that met the eligibility criteria (Fig 1). Of these, 1152 (11.8%) were missing an HIV diagnosis date and were excluded from the analysis. There were no major differences in individuals missing an HIV diagnosis date, compared to those with an HIV diagnosis date (S2 Table). Baseline characteristics of the sampled population (n = 8592, herewith referred as the sampled population) were compared to the general population started on cART between 2015HY2 and 2018HY1 (n = 358,676). There were no major differences in the sampled population, compared to general population by calendar half-year of cART initiation (2015HY2, 15.2% vs 15.6%), gender (female, 68.3% vs. 68.6%), age (15.0–24.9 years, 14.0% vs. 17.5%) and baseline CD4 T-cell count (<200 cells/mm^3^, 10.4% vs. 8.8%) respectively (S3 Table).

Overall, 8592 individuals from 38 facilities representing 38 of the 47 administrative counties in Kenya were included in the analysis. Of these, 1305 (15.2%), 1351 (15.7%), 1862 (21.7), 1318 (15.3%), 1249 (14.5%) and 1507 (17.5%) initiated cART in 2015HY2, 2016HY1, 2016HY2, 2017HY1, 2017HY2 and 2018HY1, respectively. The overall median age at cART initiation was 35.1 (IQR: 28.5–43.5) years. The majority of individuals were female (68.2%) and initiated on an EFV-based first-line regimen (72.6%). As expected, a majority of the participants were missing baseline CD4 T-cell count, increasing from 52.7% in 2015HY2 to 85.6% in 2018HY1 (Table 1).

**Table 1:** Distribution of HIV infected adults initiating combination antiretroviral therapy and eligible for the analysis using data from the national data warehouse in Kenya (2015HY2 to 2018HY1, N = 8592).

Characteristics		2015HY2	2016HY1	2016HY2	2017HY1	2017HY2	2018HY1	Overall
		(n = 1305)	(n = 1351)	(n = 1862)	(n = 1318)	(n = 1249)	(n = 1507)	(n = 8592)
**Gender**	Female	916 (70.2)	902 (66.8)	1309 (70.3)	910 (69.0)	855 (68.5)	972 (64.5)	5864 (68.2)
	Male	389 (29.8)	449 (33.2)	553 (29.7)	408 (31.0)	394 (31.5)	535 (35.5)	3728 (31.8)
**Age, years**	Median (IQR)	35.6 (28.8–43.0)	35.5 (28.8–44.2)	35.8 (29.2–44.0)	34.0 (27.6–42.5)	34.5 (27.9–43.3)	34.8 (28.6–43.9)	35.1 (28.5–43.5)
**Age group, years**	15.0–24.9	173 (13.3)	182 (13.5)	223 (12.0)	220 (16.7)	191 (15.3)	216 (14.3)	1205 (14.0)
	25.0–34.9	456 (34.9)	475 (35.2)	662 (35.6)	487 (36.9)	446 (35.7)	546 (36.2)	3072 (35.8)
	35.0–44.9	399 (30.6)	379 (28.1)	554 (29.8)	360 (27.3)	348 (27.9)	411 (27.3)	2451 (28.5)
	45.0+	277 (21.2)	315 (23.3)	423 (22.7)	251 (19.0)	264 (21.1)	334 (22.2)	1864 (21.7)
**First-line ART regimen**	NVP-based	44 (3.4)	45 (3.3)	38 (2.0)	30 (2.3)	33 (2.6)	42 (2.8)	232 (2.7)
	EFV-based	940 (72.0)	952 (70.5)	1358 (72.9)	944 (71.6)	918 (73.5)	1126 (74.7)	6238 (72.6)
	Others	85 (6.5)	89 (6.6)	123 (6.6)	70 (5.3)	89 (7.1)	130 (8.6)	586 (6.8)
	Missing	236 (18.1)	265 (19.6)	343 (18.4)	274 (20.8)	209 (16.7)	209 (13.9)	1536 (17.9)
**Baseline CD4 T-cell count (cells/mm^3^)**	<200	247 (18.9)	223 (16.5)	151 (8.1)	110 (8.3)	88 (7.0)	73 (4.8)	892 (10.4)
	200–349	173 (13.3)	135 (10.0)	106 (5.7)	76 (5.8)	57 (4.6)	45 (3.0)	592 (6.9)
	350–499	149 (11.4)	126 (9.3)	96 (5.2)	48 (3.6)	44 (3.5)	49 (3.3)	512 (6.0)
	500+	48 (3.7)	68 (5.0)	334 (17.9)	71 (5.4)	48 (3.8)	50 (3.3)	619 (7.2)
	Missing	688 (52.7)	799 (59.1)	1175 (63.1)	1013 (76.9)	1012 (81.0)	1290 (85.6)	5977 (69.6)

### Uptake of universal test-and-treat

**Fig 2:**
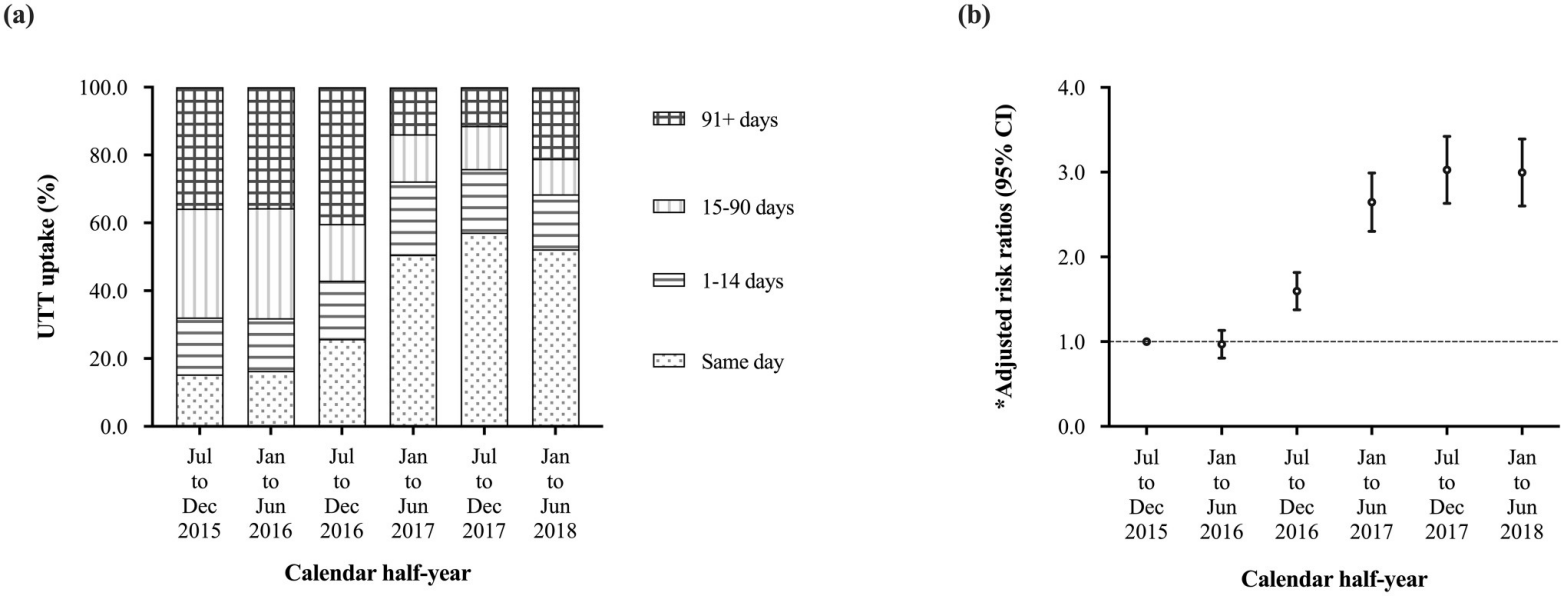
Uptake of universal test-and-treat presented as proportions of HIV infected individuals starting combination antiretroviral therapy on the same day, 1–14 days, 15–90 days and 91+ days from a HIV diagnosis using data from the national data warehouse in Kenya (2015HY2 to 2018HY1, N = 8592).

Overall, 3070 (35.7%) of individuals started cART on the same day of an HIV diagnosis. Same day HIV diagnosis and cART start increased from 15.3% prior to UTT policy implementation in 2015HY2 to 52.2% in 2018HY1. Using the 14-day threshold as per the Kenyan national guidelines, UTT uptake increased from 32.1% in 2015HY2 to 68.4% in 2018HY1 (Fig 2a).

Individuals started cART in 2018HY1 were three-fold more likely to have same day HIV diagnosis and cART initiation compared to those started cART in 2015HY2 (adjusted risk ratio, aRR: 3.0 [2.6–3.4], p<0.001), with the highest jump observed immediately after implementation of the UTT policy in mid-2016 (Fig 2b). Female gender, younger age and an EFV-based (compared to NVP-based) regimen were also independently associated with increased likelihood for same day HIV diagnosis and cART initiation (Table 2). Using the 14-day threshold as per the Kenyan national guidelines, similar observations were made; individuals started cART in 2018HY1 were two-fold more likely to start cART within 14 days of HIV diagnosis compared to those started cART in 2015HY2 (S4 Table).

**Table 2:** Factors associated with uptake of universal test-and-treat, defined as same-day HIV diagnosis and combination antiretroviral therapy start, amongst HIV infected adults using data from the national data warehouse in Kenya (2015HY2 to 2018HY1, N = 8592).

Characteristics		UTT uptake (%)	Crude risk ratio (95% CI)	p-value	Adjusted risk ratio (95% CI)	p-value
**Half-year ART initiation**	2015HY2	200/1305 (15.3)	Ref		Ref	
	2016HY1	222/1351 (16.4)	1.0 (0.8–1.2)	0.881	1.0 (0.8–1.1)	0.681
	2016HY2	480/1862 (25.8)	1.8 (1.5–2.0)	<0.001	1.6 (1.4–1.8)	<0.001
	2017HY1	667/1318 (50.6)	3.2 (2.7–3.9)	<0.001	2.6 (2.3–3.0)	<0.001
	2017HY2	714/1249 (57.2)	3.7 (3.2–4.3)	<0.001	3.0 (2.6–3.4)	<0.001
	2018HY1	787/1507 (52.2)	3.7 (3.2–4.3)	<0.001	3.0 (2.6–3.4)	<0.001
**Gender**	Female	2175/5864 (37.1)	1.1 (1.0–1.2)	0.001	1.1 (1.0–1.1)	0.002
	Male	895/2728 (32.8)	Ref		Ref	
**Age group, years**	15.0–24.9	570/1205 (47.3)	1.5 (1.4–1.6)	<0.001	1.2 (1.1–1.3)	<0.001
	25.0–34.9	1197/3072 (39.0)	1.3 (1.2–1.4)	<0.001	1.1 (1.1–1.2)	<0.001
	35.0–44.9	767/2451 (31.3)	1.1 (1.0–1.1)	0.242	1.0 (1.0–1.1)	0.383
	45.0+	536/1864 (28.8)	Ref		Ref	
**First-line ART regimen**	NVP-based	31/232 (13.4)	Ref		Ref	
	EFV-based	2203/6238 (35.3)	2.4 (1.6–3.5)	<0.001	2.0 (1.5–2.6)	<0.001
	Others	181/586 (30.9)	2.3 (1.5–3.5)	<0.001	1.8 (1.4–2.5)	<0.001
	Missing	655/1536 (42.6)	2.6 (1.7–3.9)	<0.001	1.9 (1.4–2.5)	<0.001
**Baseline CD4 T-cell count (cells/mm^3^)**	Missing	2648/5977 (44.3)	2.7 (2.2–3.4)	<0.001	1.9 (1.6–2.2)	<0.001
	<200	142/892 (15.9)	1.0 (0.8–1.4)	0.719	1.1 (0.9–1.4)	0.329
	200–349	104/592 (17.6)	1.1 (0.8–1.4)	0.674	1.1 (0.9–1.4)	0.210
	350–499	81/512 (15.8)	1.0 (0.7–1.4)	0.950	1.2 (1.0–1.5)	0.106

### Effect of universal test-and-treat on 12-months non-retention

**Fig 3:**
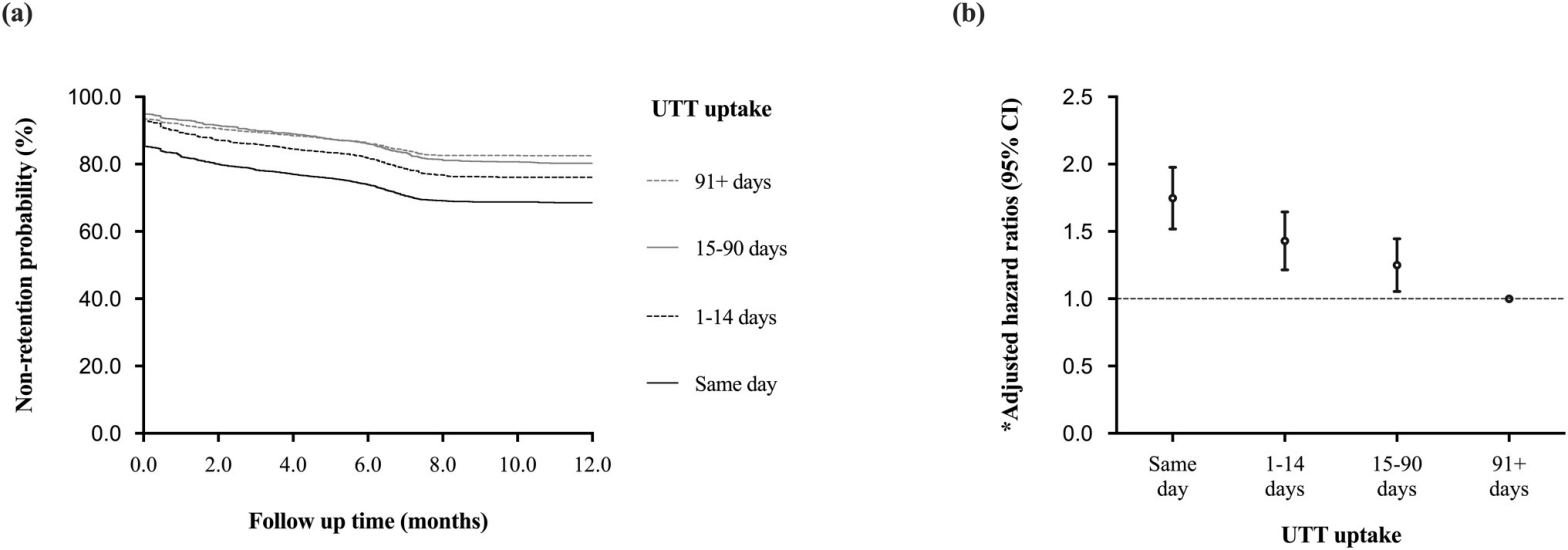
Kaplan Meier survival curve illustrating twelve months non-retention, defined as lost to follow up or dead, among of HIV infected adults by uptake of universal test and treat, defined as same day, 1–14 days, 15–90 days and 91+ days from a HIV diagnosis to combination antiretroviral therapy start, using data from the national data warehouse in Kenya (2015HY2 to 2018HY1, N = 8592).

Overall, 8592 individuals contributed 72,815 person-months observation (pmo). Of these, 2004 (23.3%) were either reported dead (n = 264, 3.1%) or LTFU (n = 1740, 20.3%) after 12-months of follow up, contributing an overall non-retention rate of 2.8 (95% CI: 2.6–2.9) per 100 pmo. Individuals initiated cART on the same day as a HIV diagnosis had the highest non-retention rate (4.0/100 pmo), followed by 1–14 days (2.7/100 pmo), 15–90 days (2.1/100 pmo) and 91+ days (1.9/100 pmo) (Fig 3a).

When compared to individuals initiated cART 91+ days after a HIV diagnosis, those initiated cART on the same day of a HIV diagnosis had the highest rate of non-retention (adjusted hazard ratio, aHR: 1.7 [95% CI: 1.5–2.0], p<0.001), followed by those initiated cART 1–14 days after a HIV diagnosis (aHR: 1.4 [95% CI: 1.2–1.6], p<0.001) and those initiated cART 15–90 days after a HIV diagnosis (aHR: 1.2 [95% CI:1.1–1.4], p = 0.006) (Fig 3b). Other factors associated with increased non-retention rates included younger age and low baseline CD4 T-cell count (Table 3).

**Table 3:** Effect of universal test-and-treat uptake, defined as same day, 1–14 days, 15–90 days and 91+ days from a HIV diagnosis to combination antiretroviral therapy start on twelve months attrition, defined as lost to follow up or dead, amongst HIV infected adults captured in the national data warehouse in Kenya (2015HY2 to 2018HY1, N = 8592).

Characteristics		d/Y (rate/100 pmo)	Crude hazard ratio (95% CI)	p-value	Adjusted hazard ratio (95% CI)	p-value
**Universal test and treat**	Same day	930/232 (4.0)	1.7 (1.5–1.9)	<0.001	1.7 (1.5–2.0)	<0.001
	1–14 days	351/130 (2.7)	1.4 (1.2–1.6)	<0.001	1.4 (1.2–1.6)	<0.001
	15–90 days	320/152 (2.1)	1.2 (1.1–1.4)	0.004	1.2 (1.1–1.4)	0.006
	91+ days	403/214 (1.9)	Ref		Ref	
**Half-year ART initiation**	2015HY2	317/112 (2.8)	Ref		Ref	
	2016HY1	327/114 (2.9)	0.9 (0.8–1.1)	0.311	0.9 (0.8–1.1)	0.316
	2016HY2	435/161 (2.7)	1.0 (0.8–1.1)	0.601	0.9 (0.8–1.1)	0.205
	2017HY1	337/106 (3.2)	1.1 (0.9–1.3)	0.311	0.8 (0.7–1.0)	0.015
	2017HY2	307/102 (3.0)	1.0 (0.9–1.2)	0.821	0.7 (0.6–0.9)	0.001
	2018HY1	281/133 (2.1)	0.7 (0.6–0.8)	<0.001	0.6 (0.5–0.7)	<0.001
**Gender**	Female	1344/500 (2.7)	1.0 (0.9–1.1)		-	-
	Male	660/228 (2.9)	Ref	0.491		
**Age group, years**	15.0–24.9	344/95 (3.6)	1.4 (1.2–1.7)	<0.001	1.3 (1.1–1.5)	0.002
	25.0–34.9	786/255 (3.1)	1.2 (1.1–1.4)	0.001	1.2 (1.0–1.3)	0.021
	35.0–44.9	521/212 (2.5)	1.1 (0.9–1.2)	0.410	1.0 (0.9–1.2)	0.667
	45.0+	353/166 (2.1)	Ref		Ref	
**First-line ART regimen**	NVP-based	31/21 (1.4)	Ref		Ref	
	EFV-based	1312/549 (2.4)	1.6 (1.1–2.3)	0.013	1.4 (1.0–2.0)	0.085
	Others	148/51 (2.9)	1.7 (1.1–2.5)	0.010	1.6 (1.1–2.4)	0.026
	Missing	513/109 (4.7)	4.5 (3.0–6.7)	<0.001	3.5 (2.3–5.3)	<0.001
**Baseline CD4 T-cell count (cells/mm^3^)**	Missing	1667/477 (3.5)	2.6 (2.0–3.5)	<0.001	2.4 (1.8–3.2)	<0.001
	<200	144/84 (1.7)	2.0 (1.4–2.7)	<0.001	1.8 (1.3–2.5)	0.001
	200–349	87/56 (1.6)	1.7 (1.2–2.3)	0.005	1.5 (1.1–2.2)	0.022
	350–499	56/49 (1.1)	1.3 (0.9–2.0)	0.132	1.3 (0.9–1.9)	0.230
	500+	50/63 (0.8)	Ref		Ref	

### Effects of universal test-and-treat on virologic non-suppression

**Fig 4:**
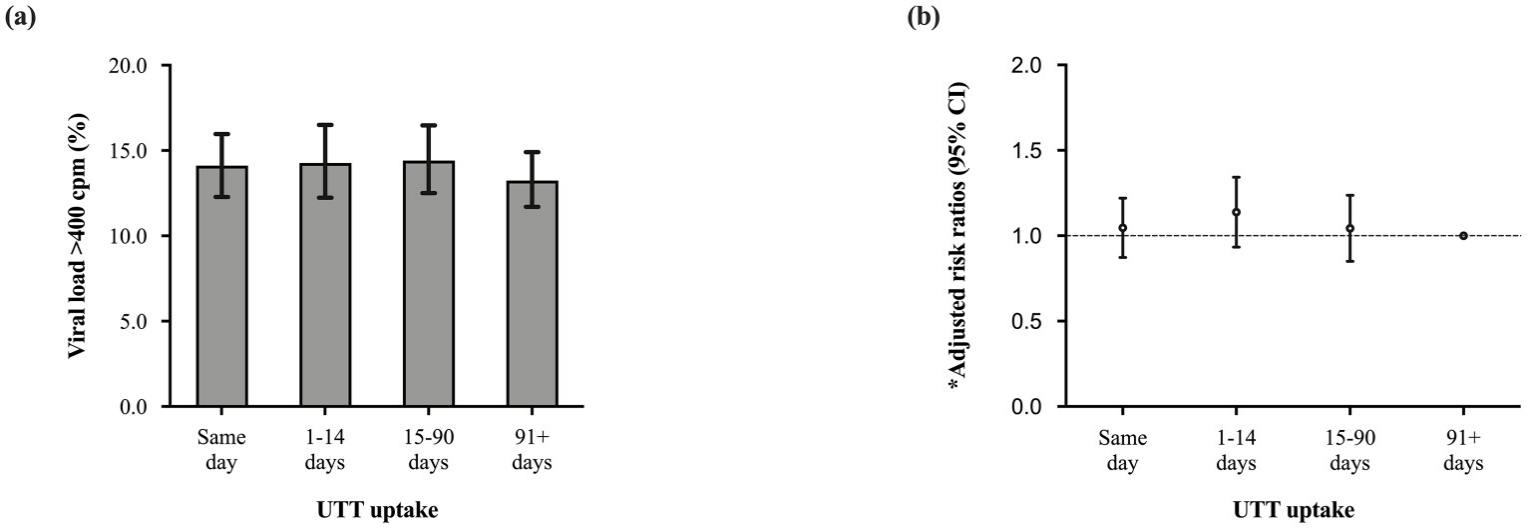
Graph showing initial virologic non-suppression (first available viral load test results of >400 copies per ml) stratified by universal test and treat, defined as same day, 1–14 days, 15–90 days and 91+ days from a HIV diagnosis to combination antiretroviral therapy start, amongst HIV infected adults starting combination antiretroviral therapy using data from the national data warehouse in Kenya (2015HY2 to 2018HY1, N = 5986).

Of the 8592 individuals included in the analysis, 5986 (69.6%) had an initial viral load test done over a median of 6.3 (IQR; 5.6–7.6) months after cART initiation. There were no significant differences in follow up time from cART initiation to initial viral load testing by UTT uptake; same day (6.3 [IQR: 5.7–7.5] months), 1–14 days (6.4 [IQR: 5.8–7.4] months), 15–90 days (6.3 [5.6–7.6] months) and 91+ days (6.2 [4.3–7.7] months). Of these, 835 (13.9%) had an initial viral load >400 copies/ml. Initial VnS for same day, 1–14 days, 15–90 days and 91+ days HIV diagnosis and cART start were 14.1%, 14.3%, 14.4% and 13.2% respectively (Fig 4a).

There was no association between UTT uptake and initial VnS. Individuals who started cART on the same day as an HIV diagnosis had the same likelihood of VnS compared to those started cART 91+ days after an HIV diagnosis (aRR: 1.0 [95% CI: 0.9–1.2], p = 0.664) (Fig 4b). Factors associated with initial VnS included younger age, low baseline CD4 T-cell count and having a viral load test done within 3 months of cART start (Table 4).

**Table 4:** Effect of universal test-and-treat, defined as same day, 1–14 days, 15–90 days and 91+ days from a HIV diagnosis to combination antiretroviral therapy start, on initial virologic non-suppression (first available viral load test results of >400) amongst HIV infected adults starting combination antiretroviral therapy and captured in the national data warehouse in Kenya (2016-HY2 to 2018-HY1, N = 5986).

Characteristics		VL >400 cpm (%)	Crude risk ratio (95% CI)	LRT p-value	Adjusted risk ratio (95% CI)	LRT p-value
**Universal test and treat**	Same day	266/1884 (14.1)	1.1 (1.0–1.3)	0.076	1.0 (0.9–1.2)	0.664
	1–14 days	154/1079 (14.3)	1.1 (0.9–1.4)	0.151	1.1 (0.9–1.3)	0.198
	15–90 days	181/1256 (14.4)	1.1 (1.0–1.3)	0.144	1.0 (0.9–1.2)	0.747
	91+ days	234/1767 (13.2)	Ref		Ref	
**Time from cART start to initial viral load test**	<3 mos	126/578 (21.8)	1.6 (1.2–2.1)	0.002	1.4 (1.2–1.8)	0.001
	3–6 mos	221/1686 (13.1)	0.9 (0.7–1.2)	0.605	0.9 (0.8–1.1)	0.479
	6–9 mos	381/2965 (12.8)	0.9 (0.7–1.2)	0.528	0.9 (0.8–1.1)	0.300
	9–12 mos	107/757 (14.1)	Ref		Ref	
**Half-year ART initiation**	2015HY2	109/877 (12.4)	Ref		-	-
	2016HY1	129/902 (14.3)	1.2 (0.9–1.5)	0.178		
	2016HY2	176/1348 (13.1)	1.1 (0.9–1.3)	0.555		
	2017HY1	137/909 (15.1)	1.2 (1.0–1.5)	0.091		
	2017HY2	112/850 (13.2)	1.1 (0.8–1.4)	0.604		
	2018HY1	172/1100 (15.6)	1.2 (1.0–1.6)	0.070		
**Gender**	Female	568/4118 (13.8)	1.0 (0.9–1.1)	0.677	-	-
	Male	267/1868 (14.3)	Ref			
**Age group, years**	15.0–24.9	123/746 (16.5)	1.4 (1.1–1.7)	0.002	1.5 (1.2–1.9)	0.001
	25.0–34.9	306/2086 (14.7)	1.3 (1.1–1.6)	0.002	1.5 (1.2–1.8)	0.001
	35.0–44.9	251/1751 (14.3)	1.3 (1.0–1.5)	0.014	1.4 (1.1–1.6)	0.002
	45.0+	155/1403 (11.0)	Ref		Ref	
**First-line ART regimen**	NVP-based	25/183 (13.7)	Ref		-	-
	EFV-based	589/4544 (13.0)	1.1 (0.8–1.6)	0.591		
	Others	63/461 (13.7)	1.2 (0.8–1.9)	0.367		
	Missing	158/798 (19.8)	1.5 (1.0–2.3)	0.059		
**Baseline CD4 T-cell count (cells/mm^3^)**	Missing	579/3950 (14.7)	1.9 (1.3–2.7)	<0.001	1.9 (1.3–2.7)	0.001
	<200	125/687 (18.2)	2.3 (1.6–3.4)	<0.001	2.5 (1.7–3.6)	<0.001
	200–349	54/442 (12.2)	1.6 (1.0–2.4)	0.033	1.5 (1.0–2.4)	0.061
	350–499	34/385 (8.8)	1.1 (0.7–1.9)	0.618	1.1 (0.7–1.9)	0.660
	500+	43/522 (8.2)	Ref		Ref	

### Comparing estimates between the sampled population and the general population

Patterns of UTT uptake and its effect on non-retention and initial VnS were similar in the general population, when compared to those observed from the sampled population. In brief and in the general population, same-day HIV diagnosis and cART start increased between 2015HY2 and 2018HY1, with the highest jump observed immediately after implementation of the UTT policy in mid-2016 (S1a Fig). Individuals initiated cART on the same day as an HIV diagnosis had the highest rate of non-retention (S1b Fig). Initial VnS rates were also comparable, with little evidence of a significant difference by UTT uptake in the general population, compared to the sampled population (S1c Fig).

## Discussion

While early cART has been shown to improve retention and virologic suppression in controlled clinical trials, much less is known about the uptake of UTT and its effect on retention and virologic suppression in ‘real life’ programmatic settings. Routine national level HIV service delivery data from Kenya suggest commendable uptake of the WHO recommended UTT policy, with a three-fold increased likelihood for same day HIV diagnosis and cART initiation over two calendar years of implementation. These findings are consistent with reports suggesting a strong impact of the UTT policy on increased uptake of early cART in Kenya and other LMIC [13, 27]. However, our findings also point towards several challenges associated with the implementation of UTT in Kenya.

First, our data demonstrates that the steady increase in UTT uptake over calendar half-years was associated with an increase in non-retention rates over the same duration. Implementation of UTT in Kenya resulted in increased numbers of people in need of cART which, in turn, may have exerted a strain on health systems and service delivery. These inherent implementation challenges, including potential inadequate human resource, were anticipated [14, 28, 29], and warrant consideration for innovative cost-effective strategies towards decongesting the increasing health system challenges associated with the implementation of UTT in Kenya.

Second, and despite evidence from randomized controlled trials (RCTs) demonstrating improved retention and virologic suppression amongst individuals on early cART [20], our findings from a routine HIV program setting demonstrate the opposite. We observed that the earlier an individual initiated cART, the higher the rate of non-retention. We also observed an impressive overall initial VnS rate of about 14%, which suggests that the country is well on its way towards achieving the UNAIDS 95-95-95 targets by the year 2030. However, we also observed a lack of an association between UTT uptake and initial VnS. Our findings are consistent with those observed from another routine setting in Nigeria which reported high rates of LTFU, poor viral load monitoring and low levels of virologic suppression amongst individuals starting cART within 14 days of HIV diagnosis [25]. Together, and in contrast with data from controlled trial settings, these findings suggest that the effectiveness of UTT on retention and virologic suppression may be much lower in routine program settings. Unlike in routine program settings, research settings offer early cART as part of a package that includes several other interventions targeting individual patients, frontline service providers, and health systems with a combined effort to facilitate early cART uptake, enhance retention, support adherence and achieve virologic suppression. These multi-pronged interventions include service providers training, condensed accelerated counselling protocols, intensified early visits schedule, short message service reminders, point-of-care diagnostics, educational packages, non-cash financial incentives and monetary incentives [1, 6, 30–33]. Our findings therefore warrant consideration of similar intervention strategies to be administered as a package alongside the implementation of UTT guidelines if it is to achieve its optimal intended benefits, including the ambitious UNAIDS 95-95-95 targets.

Third and notably, while young adults (15–24 years) had the highest odds of early cART uptake, they were also associated with the highest rates of non-retention and highest odds of initial VnS. These findings are consistent with results from other studies suggesting a high non-retention and low virologic suppression amongst the youth and young adults from HIV programs in resource limited settings. Modelling estimates suggest that youth and young adults contribute up to 40% of all new HIV infections in Kenya [34]. Our findings therefore suggest that whilst the youth are initially ready to start cART immediately after HIV diagnosis, individual level challenges including denial, inadequate preparedness to early cART, stigma and disclosure may be potential barriers to retention and achieving virologic suppression [35]. These findings point to the need for a customised UTT package of care that will also address youth-specific challenges.

A major strength of our analysis is the use of routine service delivery data from health facilities located in 38 of the 47 counties in Kenya. However, use of routine service delivery data does have innate limitations. First, our convenient sampling strategy may have potentially introduced selection bias. However, a supplementary analysis of data from the general population, when compared to the sampled population, demonstrates comparable patterns on uptake of UTT, non-retention and initial VnS endpoints. The sampling strategy may also have resulted to inclusion of sites that are better functioning. Possibility that the lack of difference in initial VnS may be due to well-functioning program prior to implementation of UTT can therefore not be ruled out. Second, additional individual level data (including pre-ART counseling, disclosure, involvement in support groups, adherence to treatment, incentives/motivation) and facility level data (number/cadre of service providers, continued training of service providers, availability of point-of-care diagnostics) would have enabled control for possible confounding. However, this was not possible as these variables were not systematically and consistently documented. Third, more than half the individuals were missing baseline CD4 T-cell count data, which made it impossible to completely control for disease severity at cART initiation in this analysis. While a limitation, this is not surprising as CD4-T cell count monitoring was discouraged after the introduction of the UTT policy in Kenya. Last, our definition of initial VnS was based on the first available viral load test result. As per the Kenyan guidelines, the first viral load test is recommended at 6–12 months after cART initiation. As such, we were not able to compare timing of virologic suppression achieved after UTT uptake. However, we were able to stratify VnS by duration from cART into 3-month follow up periods. Compared to those with a viral load test done >9 months, individuals tested <3 months after cART had higher likelihood of VnS; likely because shorter follow up time was not sufficient to achieve virologic suppression.

## Conclusions

Limitations above not-withstanding, our findings suggest commendable uptake of the UTT policy in Kenya. However, despite evidence from controlled studies suggesting that early cART improves retention and virologic suppression, our findings suggest poor twelve-months retention and lack of an association with initial VnS in routine real-life program settings. This difference in observations may largely be attributed to multiple other interventions co-implemented alongside early cART in controlled studies. Our findings therefore warrant consideration for multi-component program interventions targeting individuals, service providers and health systems, to complement implementation of the UTT policy towards achieving epidemic control in Kenya.

## Supporting information

S1 Table An outline of facilities that met the eligibility criteria (lowest proportion missing date of HIV diagnosis and a viral load test done within 12 months of combination antiretroviral therapy initiation) from the 38 facilities from 38 counties contributing to the national data warehouse in Kenya and included in the analysis (2015-HY2 to 2018-HY1). (TIF)

S2 Table Comparison of characteristics between HIV infected individuals starting combination antiretroviral therapy between 2015HY2 to 2018HY1 but had a missing date of HIV diagnosis and those with a date of HIV diagnosis (N = 9,744). (TIF)

S3 Table Comparison of characteristics between HIV infected individuals included in the final analysis (N = 8,592) and those that started combination antiretroviral therapy between 2015HY2 to 2018HY1 from the general population from all facilities in Kenya (N = 358,676). (TIF)

S4 Table Factors associated with uptake of universal test and treat, defined as <14 days from HIV diagnosis and combination antiretroviral therapy start, amongst HIV infected adults using data from the national data warehouse in Kenya (2015-HY2 to 2018-HY1, N = 8592). (TIF)

S1 Fig (a) Uptake of universal test-and-treat presented as proportions of HIV infected individuals starting combination antiretroviral therapy on the same day, 1–14 days, 15–90 days and 91+ days from a HIV diagnosis; (b) Twelve months non-retention, defined as lost to follow up or dead, among of HIV infected adults by uptake of universal test-and-treat; and (c) initial virologic non-suppression (first available viral load test results of >400 copies per ml) between the sampled population and the general population of individuals started cART between 2015HY2 and 2018HY1 in Kenya (N = 358,676). (TIF)
