# Supplementary figures and images for: Uptake and effect of universal test-and-treat on twelve months retention and initial virologic suppression in routine HIV program in Kenya
Source: PLoS One. 2022 Nov 22;17(11):e0277675. doi: 10.1371/journal.pone.0277675 (PMC9681077; doi:10.1371/journal.pone.0277675)

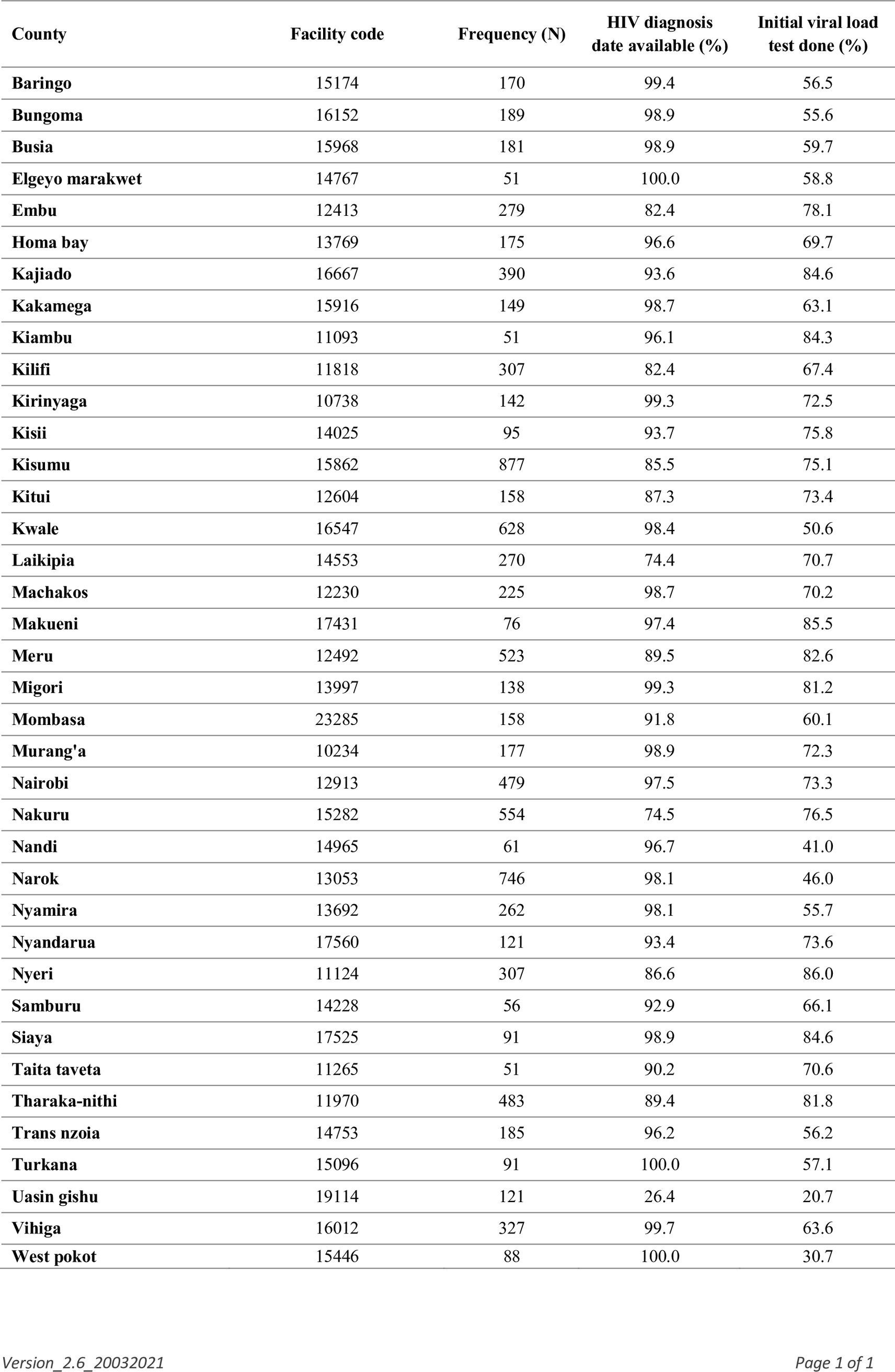

Supplement: S1 Table — (TIF) [file pone.0277675.s001.tif]

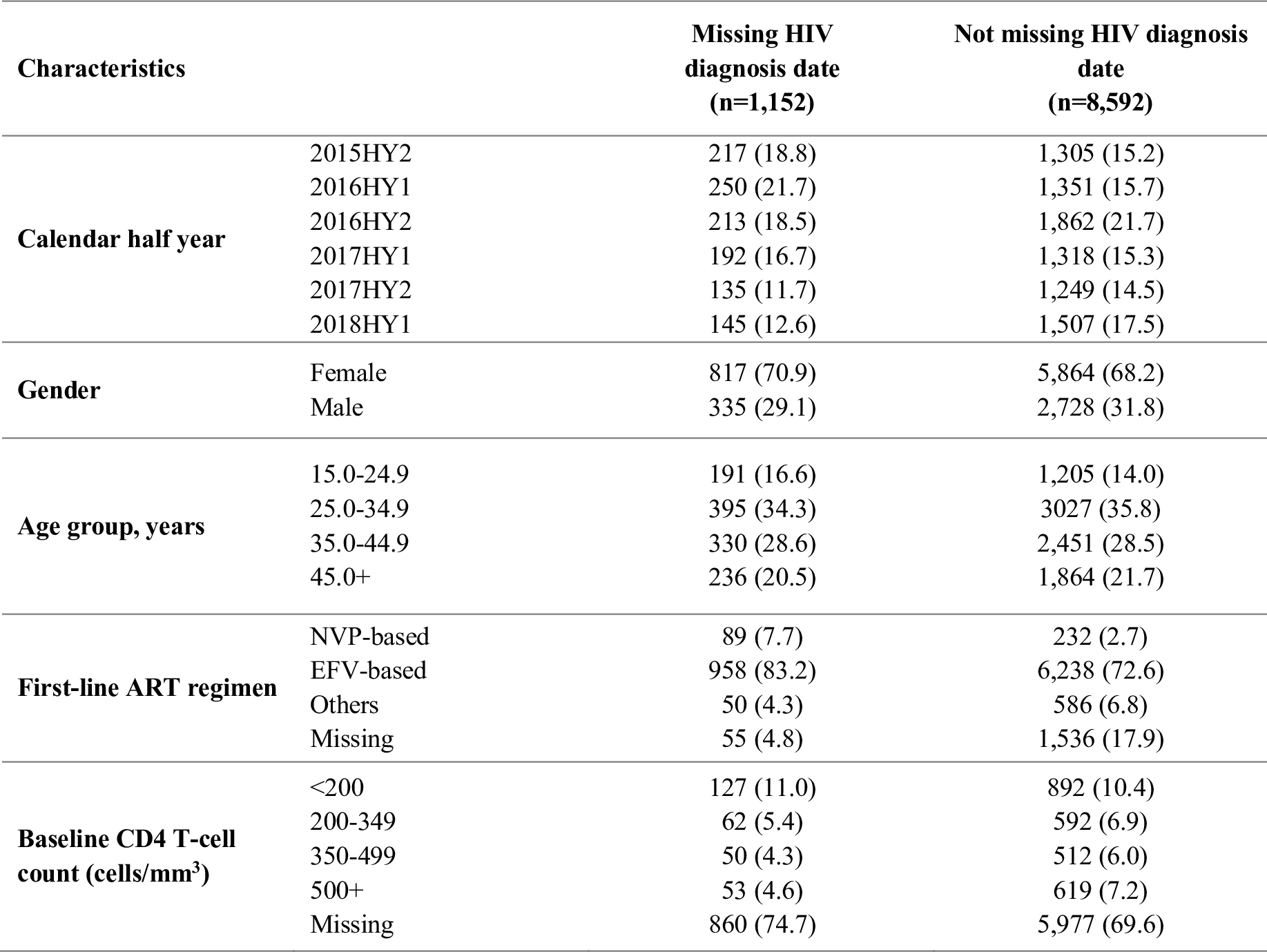

Supplement: S2 Table — (TIF) [file pone.0277675.s002.tif]

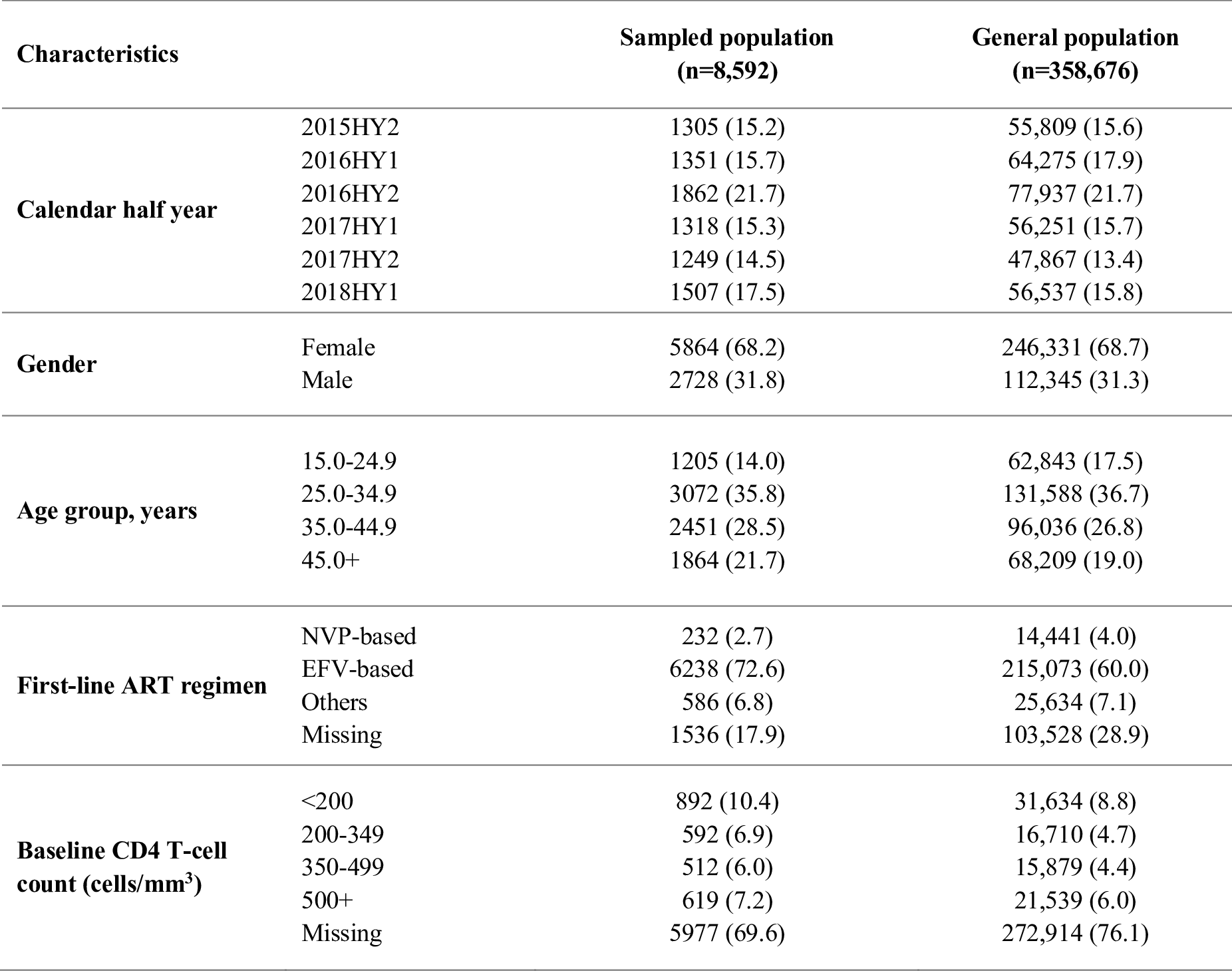

Supplement: S3 Table — (TIF) [file pone.0277675.s003.tif]

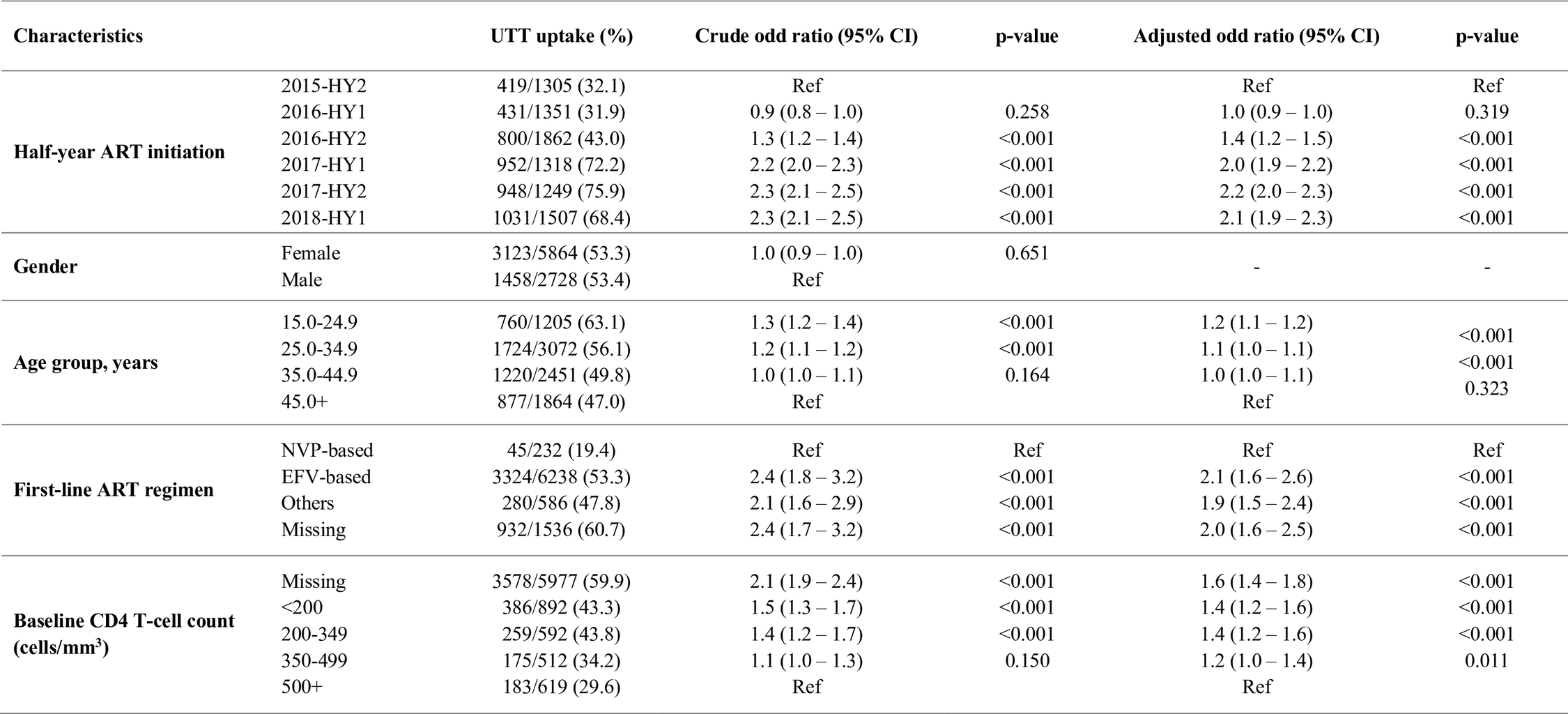

Supplement: S4 Table — (TIF) [file pone.0277675.s004.tif]

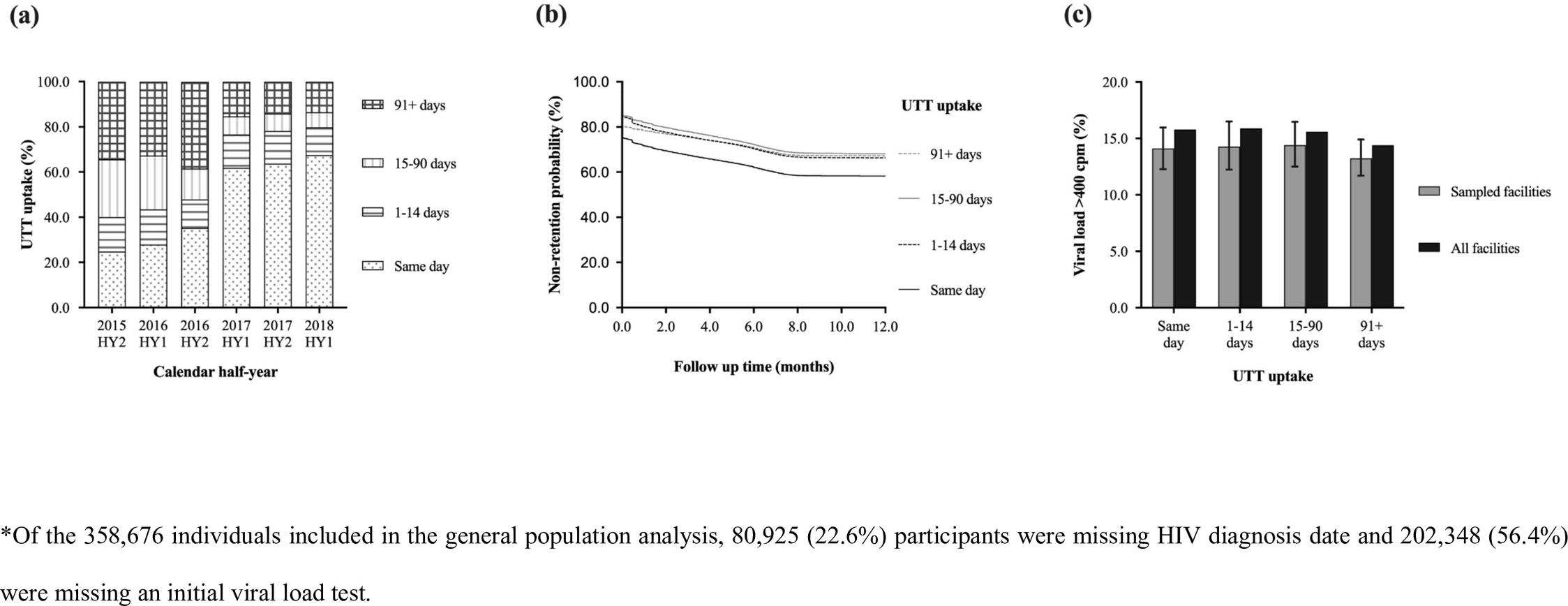

Supplement: S1 Fig — (TIF) [file pone.0277675.s005.tif]
